# Improving Adjuvant Hormone Therapy Use in Medicaid Managed Care–Insured Women, New York State, 2012–2014

**DOI:** 10.5888/pcd13.160185

**Published:** 2016-09-01

**Authors:** Victoria L. Wagner, Wei Jing, Francis P. Boscoe, Maria J. Schymura, Patrick J. Roohan, Foster C. Gesten

**Affiliations:** Author Affiliation: Wei Jing, Patrick J. Roohan, Foster C. Gesten, Office of Quality and Patient Safety, New York State Department of Health, Albany, New York; Francis P. Boscoe, Maria J. Schymura, New York State Cancer Registry, New York State Department of Health, Albany, New York.

## Abstract

**Introduction:**

In 2010, national guidelines recommended that women with nonmetastatic, hormone receptor–positive breast cancer take adjuvant hormone therapy for 5 years. As results from randomized clinical trials became available, guidelines were revised in 2014 to recommend 10 years of therapy. Despite evidence of its efficacy, low initiation rates have been documented among women insured by New York State Medicaid. This article describes a coordinated quality improvement pilot conducted by a state department of health and Medicaid managed care plans to engage women in guideline-concordant adjuvant hormone therapy.

**Methods:**

Women enrolled in Medicaid managed care with nonmetastatic, hormone receptor–positive breast cancer and who had surgery from May 1, 2012, through November 30, 2012, were identified using linked Medicaid and Cancer Registry data. Adjuvant hormone therapy status was determined from Medicaid pharmacy data. Contact information for nonadherent women was supplied to health plan care managers who conducted outreach activities. Adjuvant hormone therapy status in the 6 months following outreach was evaluated.

**Results:**

In the 6 months postoutreach, 61% of women in the contacted group filled at least 1 prescription, compared with 52% in the noncontacted group. Among those with at least 1 filled prescription, 50% of the contacted group were adherent, compared with 25% in the noncontacted group.

**Conclusion:**

This pilot suggests outreach conducted by health plan care managers, facilitated by linked Medicaid and Cancer Registry data, is an effective method to improve adjuvant hormone therapy initiation and adherence rates in Medicaid managed care–insured women.

## Introduction

Breast cancer is the most commonly diagnosed cancer in women in the United States and has the second highest death rate (1–5). In 2013 in New York State (NYS), 15,680 women were diagnosed with breast cancer, and 2,576 died (6). Roughly 70% of breast cancers are hormone receptor positive (HR-positive) (ie, the tumor’s growth is promoted by estrogen, progesterone, or both) (7,8). Adjuvant hormone therapy (AHT) works either by lowering hormone levels or blocking the action of hormone on tumor cells, and it reduces annual recurrence rates by approximately 40% as well as improves survival rates (9–11). Because results from randomized clinical trials demonstrate additional benefits from longer courses of treatment, national guidelines in 2014 recommended extending the use of AHT for women with nonmetastatic, HR-positive breast cancer from the previously recommended 2010 guideline of 5 years to 10 years, starting within a year of diagnosis (12). Despite this change in recommendation, suboptimal use of AHT is documented, particularly among low-income, minority, and young women (13–18).

NYS has one of the largest Medicaid programs in the country, serving a low-income, largely minority population. In 2013, more than 5.3 million New Yorkers were insured by Medicaid (19). Previous investigation into AHT use in NYS’s Medicaid population indicated that just 68% of women diagnosed from 2004 through 2006 initiated AHT within 1 year of diagnosis, and 58% of women who initiated AHT were adherent in the following year (20). These findings are consistent with those of previous studies (13,21).

Given the need to improve AHT use by NYS Medicaid-insured women and the ability to identify those who are not receiving AHT by linking their Medicaid data with NYS Cancer Registry (NYSCR) data, the NYS Department of Health implemented a quality improvement pilot. This article describes the coordinated, quality-improvement pilot conducted to engage women in guideline-concordant AHT care.

## Methods

### Population

NYS Medicaid encounter data (ie, health service use data submitted by managed care plans) as well as Medicaid claim and eligibility data were used to identify women aged 21 to 64 years with a breast cancer surgery (captured using breast cancer diagnosis codes in conjunction with surgical procedure codes) in the 7 month period from May through November 2012. Because this pilot was designed to target women with newly diagnosed breast cancer, women with a breast cancer surgery in the preceding 5 years were eliminated from the cohort. Medicaid data on breast cancer surgery were then linked to NYSCR data to confirm the breast cancer diagnosis and to extract information on stage of disease (local, regional, or metastatic) and tumor HR status. The cohort was further restricted to exclude women who were also Medicare beneficiaries (called “dual-eligibles”) because Medicare pharmacy data were not available. The final cohort comprised women with nonmetastatic, HR-positive breast cancer who were enrolled in one of 8 Medicaid managed care (MMC) plans that volunteered to participate in the pilot. This cohort represented approximately half (8 of 17) of the mainstream MMC plans in NYS and covered all regions of the state. 

NYS Medicaid pharmacy encounter data were used to determine AHT status. Women captured in 1 of the following 3 groups were considered eligible for outreach: *AHT noninitiated*, women with no filled AHT prescriptions from April 2012 through the date of the data pull (August 7, 2013); *AHT nonadherent*, women with a medication possession ratio of less than 0.8 (sum of days’ supply divided by the difference between last and first fill dates); and *AHT discontinued*, women with no filled prescriptions from April 2013 through the data pull. We also stratified data according to race/ethnicity (white, Hispanic, black, Asian/Pacific Islander, or Native American/other/unknown) and region of residence (New York City or elsewhere in NYS).

### Outreach

Written outreach materials were developed collaboratively with health plans and available to care managers in both English and Spanish (Appendix). Before commencing telephone outreach to patients, health plans sent letters to patients’ physicians (preferably oncologists if one was identified in encounter data). Physician letters had a dual-purpose; they were sent as a courtesy to notify physicians that the health plans would be contacting their patients, and they also requested that physicians contact health plan staff if a patient should not be called because of extenuating circumstances. After the physician letters were sent, health plans sent letters to patients informing them that a representative from the health plan would be calling to discuss their recent treatment for breast cancer. Health plan care managers made up to 5 attempts to contact each patient by telephone. To guide their conversation with patients, care managers used a telephone script that addressed current guidelines for AHT use, provided assurance that the cost of AHT was covered by health plans, and offered to help women talk with their doctors about AHT.

To facilitate outreach, the following information was extracted from Medicaid data systems and supplied to health plans: patient name, address, telephone number, date of birth, and AHT status; oncologists’ National Provider Identifier code; and an indicator to identify Spanish-speaking women. A web-based data exchange platform was used to securely transfer data to health plan staff and collect information on contact attempts, including outreach completion status and date.

For the analyses, we defined 3 groups: 1) women who met our inclusion criteria but who were AHT adherent at the start of activities, 2) women who were contacted and completed the telephone script with care managers, and 3) women whom care managers were unable to contact or who refused to complete all components of the telephone script.

Ideally, the evaluation period would commence on the date that outreach was completed; however, because of variation in health plan roll-out of pilot activities and challenges contacting women, outreach completion dates ranged from September through November 2013. Additionally, although health plan care managers submitted outreach completion dates via the web-based data exchange platform for women contacted by telephone, a similar intervention start date was not available for the 2 control groups (those who were AHT adherent and those who were not contacted). To compare the 3 groups, the median outreach completion date (September 25, 2013) was used as the standard start date for evaluating AHT outcomes for all 3 groups. To validate our results, we compared 2 sets of analyses, 1 using the actual outreach completion date and the other using the median outreach completion date. Results were the same (data not shown), suggesting that this was an appropriate approach.

### Outcome assessment and statistical analysis

NYS Medicaid data were used to identify AHT prescriptions filled within 6 months of the median outreach completion date. The proportion of women with at least 1 filled AHT prescription after September 25, 2013, was calculated. The AHT possession ratio was also calculated as the sum of days’ supply of AHT prescriptions from September 25, 2013, through March 23, 2014, divided by 180. Women were considered AHT adherent if the AHT possession ratio was greater than or equal to 0.8.

Fisher’s exact and χ^2^ tests were used to assess significance in demographic distributions between contacted and noncontacted groups. One-tailed *t* tests and 1-tailed Fisher’s exact tests were used to determine significant differences in AHT use postintervention. Analyses were performed using SAS 9.4 and SAS Enterprise Guide 6.1 (SAS Institute, Inc). Significance was set at *P* < .05. This project was granted exempt status by the New York State Department of Health’s institutional review board.

## Results

A total of 1,614 women who had breast cancer surgery from May 1, 2012, through November 30, 2012, were identified. Among these women, 255 were aged 21 to 64 years, were not dual-eligible, had been diagnosed with nonmetastatic, HR-positive breast cancer, and were enrolled in a participating health plan as of July 2013. Within this cohort, 81 (32%) women required outreach; their AHT status before outreach was as follows: 46 (57%) were AHT noninitiated, 19 (23%) were AHT nonadherent, and 16 (20%) were AHT discontinued. The remaining 174 women (68%) were AHT adherent and were not targeted for outreach.

Of the 81 women identified for outreach, 3 (4%) women were disenrolled from MMC between the release of data and the start of outreach activities, 42 (52%) were successfully contacted by telephone, and 36 (44%) could not be reached by telephone or did not complete the telephone script. As of March 2014, 163 (94%) of the AHT adherent group, 31 (86%) of the noncontacted group, and 36 (86%) women contacted were still enrolled in NYS MMC as non–dual-eligible.

Race/ethnicity proportions did not differ significantly (*P* = .87), with more than one-third of the women in each group white (Table 1). Among women in the contacted group, 47% were aged 50 to 64 years, whereas 68% were in the noncontacted group; however, the difference was not significant (*P* = .16). Most women in both groups resided in New York City (*P* = .89). No difference in proportion between the groups was noted with respect to stage of disease; most women in both groups had localized breast cancer (*P* = .89). The AHT adherent group served as a positive control for the adherence after the outreach, and data for this group are also presented (Table 1).

**Table 1:** Characteristics of Women in a Pilot Study to Improve Adjuvant Hormone Therapy Use Among Those With Breast Cancer^a^, by Cohort, 8 Medicaid Managed Care Health Plans in New York State, 2012–2014

Characteristic	Study Cohort			Total No. (N = 230)	*P* Value^b^
	AHT Adherent (n = 163)	AHT Nonadherent			
		Contacted (n = 36)	Not Contacted (n = 31)		
	No. (%)				
**Race/ethnicity**					
White	37 (23)	13 (36)	11 (35)	61	.87^b^
Hispanic	54 (33)	9 (25)	7 (23)	70	
Black	30 (18)	9 (25)	7 (23)	46	
Asian/Pacific Islander	28 (17)	4 (11)	3 (10)	35	
Native American/other/unknown	14 (9)	1 (3)	3 (10)	18	
**Age group, y**					
50–64	95 (58)	17 (47)	21 (68)	133	.16^d^
40–49	53 (33)	13 (36)	5 (16)	71	
21–39	15 (9)	6 (17)	5 (16)	26	
**Region of residence**					
New York City	123 (75)	25 (69)	22 (71)	170	.89^d^
Elsewhere in New York State	40 (25)	11 (31)	9 (29)	60	
**Cancer stage**					
Local	105 (64)	25 (69)	22 (71)	152	.89^d^
Regional	58 (36)	11 (31)	9 (29)	78	

Abbreviation: AHT, adjuvant hormone therapy.

a Women who were not dually eligible for Medicare, who were aged 21 to 64 years with diagnosed nonmetastatic hormone receptor–positive breast cancer, and who had breast cancer surgery from May 1, 2012, through November 30, 2012.

b Compares distribution in the noncontacted and contacted groups.

c Calculating using Fisher’s exact test.

d Calculating using χ^2^ test.

Among patients who were successfully contacted, 22 of 36 (61%) initiated AHT within 6 months of outreach (Table 2). This number was higher than for the group not successfully contacted; only 16 of 31 patients (52%) had initiated AHT. Among those with at least 1 filled AHT prescription, the contacted group had an average of 4.4 AHT prescriptions filled during the 6-month follow-up period, compared with 3.4 in the unable-to-contact group (data not shown). Although the adherence rate for the contacted group (50%) was not as high as that for the AHT adherent group (86%), it was higher than that for the nonadherent/noncontacted group (25%). Outreach status was marginally associated with adherence after outreach (*P* = .11).

**Table 2:** AHT Initiation and Adherence 6 Months Postoutreach, by Cohort, Pilot Study to Improve AHT Use by Women With Breast Cancer in 8 New York State Medicaid Managed Care Health Plans, 2014

Characteristic	Study Cohort			Total No.	*P* Value^a^
	AHT Adherent	AHT Nonadherent/			
		Contacted	Not Contacted		
**At least 1 filled AHT prescription post-September 25, 2013**					
Yes, no. (%)	156 (96)	22 (61)	16 (52)	194	.20^b^
No, no. (%)	7 (4)	14 (39)	15 (48)	36	
Total no.	163	36	31	230	—
**AHT adherent^c^ postoutreach**					
Yes, no. (%)	134 (86)	11 (50)	4 (25)	149	.11^d^
No, no. (%)	22 (14)	11 (50)	12 (75)	45	
Total no.	156	22	16	194	—

Abbreviations: — , not applicable; AHT, adjuvant hormone therapy.

a Compares adherence rates of the noncontacted and contacted groups.

b Calculated using χ^2^ test.

c Data limited to women with at least 1 filled AHT prescription post-September 25, 2013.

d Calculated using Fisher’s exact test.

We also compared the adherence rates in different subgroups (Table 3). Among women with a preoutreach status of AHT noninitiated who were contacted, 60% were adherent after outreach, compared with 37% in the noncontacted group. Higher adherence rates were observed among white, black and Asian/Pacific Islander women, as well as across all strata of age, region of residence, cancer stage, and preoutreach status.

**Table 3:** Comparison of Adherence Rates Postoutreach, by Selected Demographics and Cohort, Among Women With at Least 1 Filled AHT Prescription Post-September 25, 2013, Pilot Study to Improve AHT Use by Women with Breast Cancer in 8 New York State Medicaid Managed Care Health Plans, 2014

Patient Demographic/Disease Status	Adherence Rate Postoutreach		
	AHT Adherent	AHT Nonadherent	
		Contacted	Not Contacted
	No. (%)		
**Race/ethnicity**			
White	29 (83)	6 (75)	1 (14)
Hispanic	48 (91)	1 (17)	2 (50)
Black	24 (83)	3 (43)	0
Asian/Pacific Islander	22 (81)	1 (100)	1 (50)
Native American/other/unknown	11 (92)	0	0
**Age group, y**			
50–64	79 (87)	5 (50)	3 (30)
40–49	42 (84)	4 (44)	1 (33)
21–39	13 (87)	2 (67)	0
**Region of residence**			
New York City	102 (86)	8 (53)	3 (25)
Elsewhere in New York State	32 (87)	3 (43)	1 (25)
**Cancer stage**			
Local	92 (90)	5 (36)	2 (18)
Regional	42 (78)	6 (75)	2 (40)
**Preoutreach status**			
AHT noninitiated	0	6 (60)	3 (37)
AHT discontinued	0	3 (50)	0
AHT nonadherent	0	2 (33)	1 (14)
**Total**	134 (86)	11 (50)	4 (25)

Abbreviation: AHT, adjuvant hormone therapy.

## Discussion

Nearly one third (32%) of MMC-insured women with nonmetastatic, HR-positive breast cancer were not AHT adherent and met our eligibility criteria. Results of the pilot suggest a benefit from health plan outreach, because women who were contacted were more likely to initiate and adhere to AHT, although the differences were not significant. Subpopulations such as white, black and Asian/Pacific Islander, as well as all strata of age group, region of residence, cancer stage and preoutreach status showed improvement in AHT adherence in the contacted group compared with the noncontacted group, suggesting that the outreach may be effective for diverse demographic groups. Among all races/ethnicities, Native American/other/unknown women had a high adherence rate before outreach (77.8%), as did Hispanic women (77.1%) (Table 1). These rates are comparable with rates reported in the literature of 70% to 85% (15,21–24). However, white and black women lagged behind with preoutreach AHT adherence rates of 60.7% and 65.2%, respectively. Given the observed disparities within our cohort of MMC-insured women, it makes sense that the greatest gains made by our outreach were among the groups with the lowest AHT adherence at baseline.

Our study has limitations. Medicaid pharmacy data indicate that a prescription was filled and picked up by the patient; it does not, however, indicate that the patient took the medication as prescribed. Also, this was a small-scale pilot, in which half of MMC plans participated. As such, the number of women included in the pilot and available for analysis was small. Because of our small study population, what appear to be meaningful differences between groups were not statistically significant. We did not conduct a randomized control trial; our testing and negative control groups were categorized on the basis of whether we could successfully contact them, which may have introduced bias between the 2 groups.

Although the number of breast cancers diagnosed in the NYS Medicaid population is small, this project (by encouraging MMC-insured women to initiate and adhere to AHT) has the potential to prevent cancer recurrence and save lives. For these reasons and based on the initial, positive findings presented here — in addition to the low cost of the project — the NYS Department of Health is moving forward to implement this pilot statewide in all mainstream MMC plans. Additionally, as NYS moves to develop an all-payer database (APD), the information contained within the APD would allow this project to expand and include Medicare and commercially insured women.

## Appendix. Outreach Materials Used in the Pilot.

This file is available for download as a Microsoft Word document.
