# Supplementary material for: Improving Adjuvant Hormone Therapy Use in Medicaid Managed Care–Insured Women, New York State, 2012–2014
Source: Prev Chronic Dis. 2016 Sep 1;13:E120. doi: 10.5888/pcd13.160185 (PMC5008863; doi:10.5888/pcd13.160185)
Supplement: Supplementary file 1 [file 16_0185_Appendix.docx]

Appendix 1: Outreach Materials Used in the Pilot

**Letter to Provider**

Dear Dr._____, (Oncologist, if none, then PCP)

[Health Plan Name], in conjunction with the New York State Department of Health, is implementing a quality improvement project to increase adjuvant hormone therapy (AHT) usage among Medicaid managed care enrolled women who have been diagnosed with non-metastatic, hormone-receptor positive breast cancer. Research suggests that these women would benefit greatly from initiating AHT within one year of diagnosis. New York State Medicaid pharmacy data indicate that your patient, _____, either has not filled a prescription for AHT or has not been filling prescriptions regularly. Care managers from our health plan will be calling her to discuss the importance of AHT, to re-engage her in care and to conduct a short telephone survey. We ask for your cooperation in ensuring she gets AHT. If there are any reasons why you feel we should not contact this patient, or if there is anything you would like us to know or to discuss with us, please let us know within the next week at ______________. Ask for:_______________________________________

Sincerely,

[Health Plan Name]

cc: Enrollee’s PCP

Enrollee’s Surgeon

# Introduction Letter to Enrollee

Dear Ms. _________,

We at [Health Plan Name] care about your health and want to make sure you are getting excellent health care. According to our information, you were recently treated for breast cancer. [Health Plan Name] is working with the New York State Department of Health to make sure that all women in New York with breast cancer get the right care at the right time. I am a nurse and I would like to speak with you to find out how you are doing and to see if there is anything [Health Plan Name] can do to help you get the best care possible. I hope you can take a few minutes to speak with me. I will be calling you in the next week. Should you prefer to call me, I can be reached at [Phone Number].

I look forward to speaking with you soon.

Sincerely,

____________________Health Plan Care Manager

# Telephone Script for Care Management

Please Note: HIGHLIGHTING used to identify areas where plans may want to customize the script

Hello, I am a nurse from <HEALTH PLAN NAME> my name is <NURSE>, may I please speak with <MEMBER>?

**MEMBER ANSWERS:** Hi, <MEMBER>, I am calling to talk about your health care, did you receive a letter from me last week? *Pause*

**SOMEONE ELSE ANSWERS AND GETS MEMBER*:*** Hello, <MEMBER>, my name is <NURSE>, I am a nurse from <HEALTH PLAN NAME> and I’m calling to talk about your health care. Did you receive a letter from me last week? *pause*

**“YES”:** Good, then you know I am calling because <HEALTH PLAN NAME> is working with the New York State Department of Health to ensure that women with breast cancer get the right care at the right time. We recently learned you have been treated for breast cancer and I want to find out how you are doing. How is it going? *(Pause)*

**“NO”:** OK, well, I am calling because <HEALTH PLAN NAME> is working with the New York State Department of Health to ensure that women with breast cancer get the right care at the right time. We recently learned you have been treated for breast cancer and I want to find out how you are doing. How is it going? *(Pause)*

*Nurse to respond sympathetically to member and to note concerns raised. If identified issues require simple intervention by health plan (e.g. change of PCP, transportation, pharmacy card, etc.) the caller should be transferred for assistance as appropriate at the end of the call. If more extensive care coordination or case management is appropriate a case should be opened at the end of the call.*

I have some information that might be helpful for you. Experts in treating breast cancer recommend that women with breast cancer like yours take a certain type of medicine daily for 5 years. The medicine is called adjuvant hormone therapy or AHT. There are several different brands of AHT. Prescriptions for AHT are covered by <HEALTH PLAN NAME> which means that you don’t need to pay for this medication.

Can I help you talk with your doctors to find out if AHT would help you? *(pause. If member asks, names of AHT brands include: Tamoxifen,* [*anastrazole*](http://www.cancer.gov/cancertopics/druginfo/anastrozole) *(Arimidex®),* [*exemestane*](http://www.cancer.gov/cancertopics/druginfo/exemestane) *(Aromasin®),* [*letrozole*](http://www.cancer.gov/cancertopics/druginfo/letrozole) *or (Femara®).*

**“YES”:** Good, It would help if I understood a little better how you have been treated so far.

**“NO”:** OK, I want to help you to get the best care, maybe it would help me understand a little better if I knew how you have been treated so far.

******Optional questions for care managers to ask to assist in getting the member back into care******

My records show that <DR PCP> is your regular doctor, is that right? *(get corrected name if needed.)* Has he (or she) been helping or talking with you about your cancer treatment? *Make note of response.*

Are you taking any pills for breast cancer?

**“YES”:** Who is the doctor who is writing the prescription for the pills you are taking? *Make note of prescribing doctor for follow-up.* What is the name of the medicine? *Make note of response*

**“NO”:** Did any doctor ever say that you should or could take some pills for cancer?

**“YES”:** Do you remember that doctor’s name? *Make note of prescribing doctor for follow-up.*

**“NO”:** Did you have surgery for your breast cancer? Can you tell me the name of your surgeon? *If not: Where was the surgery done? Make note of surgeon and/or facility for follow-up.*

Have you had any other treatment for breast cancer? Radiation? Chemotherapy (cancer medication)? Do you know the names of those doctors? *If not, where was the treatment done? Make note of doctors and facilities for follow-up.*

Thank you. This information helps. May I get in touch with your doctors to see if they think that AHT would be a good treatment for you? If it is, I will help you make an appointment to discuss it with your doctor. Is there anything else I can answer or do for you now? *(Transfer member if needed for PCP change, pharmacy card, transportation, open CM case if needed.)*
